# Enhanced fluorescent properties of an OmpT site deleted mutant of Green Fluorescent Protein

**DOI:** 10.1186/1475-2859-9-26

**Published:** 2010-04-29

**Authors:** Shardul S Salunkhe, Veena A Raiker, Sachin Rewanwar, Prakash Kotwal, Avijeet Kumar, Sriram Padmanabhan

**Affiliations:** 1 Lupin Limited, Biotechnology R & D, Gat #1156, Ghotawade Village, Mulshi Taluka, Pune-411042, India; 2 Project Trainee, M. Tech (Int) Biotechnology, Dr. D.Y. Patil Biotechnology and Bioinformatics Institute, Pune, India

## Abstract

**Background:**

The green fluorescent protein has revolutionized many areas of cell biology and biotechnology since it is widely used in determining gene expression and for localization of protein expression. Expression of recombinant GFP in *E. coli *K12 host from pBAD24M-GFP construct upon arabinose induction was significantly lower than that seen in *E. coli *B cells with higher expression at 30°C as compared to 37°C in *E. coli *K12 hosts. Since OmpT levels are higher at 37°C than at 30°C, it prompted us to modify the OmpT proteolytic sites of GFP and examine such an effect on GFP expression and fluorescence. Upon modification of one of the two putative OmpT cleavage sites of GFP, we observed several folds enhanced fluorescence of GFP as compared to unmodified GFPuv (Wild Type-WT). The western blot studies of the WT and the SDM II GFP mutant using anti-GFP antibody showed prominent degradation of GFP with negligible degradation in case of SDM II GFP mutant while no such degradation of GFP was seen for both the clones when expressed in BL21 cells. The SDM II GFP mutant also showed enhanced GFP fluorescence in other *E. coli *K12 OmpT hosts like *E. coli *JM109 and LE 392 in comparison to WT GFPuv. Inclusion of an OmpT inhibitor, like zinc with WT GFP lysate expressed from an *E. coli *K12 host was found to reduce degradation of GFP fluorescence by two fold.

**Results:**

We describe the construction of two GFP variants with modified putative OmpT proteolytic sites by site directed mutagenesis (SDM). Such modified genes upon arabinose induction exhibited varied degrees of GFP fluorescence. While the mutation of K79G/R80A (SDM I) resulted in dramatic loss of fluorescence activity, the modification of K214A/R215A (SDM II) resulted in four fold enhanced fluorescence of GFP.

**Conclusions:**

This is the first report on effect of OmpT protease site modification on GFP fluorescence. The wild type and the GFP variants showed similar growth profile in bioreactor studies with similar amounts of recombinant GFP expressed in the soluble fraction of the cell. Our observations on higher levels of fluorescence of SDM II GFP mutant over native GFPuv in an OmpT^+ ^host like DH5α, JM109 and LE392 at 37°C reiterates the role played by host OmpT in determining differences in fluorescent property of the expressed GFP. Both the WT GFP and the SDM II GFP plasmids in *E. coli *BL21 cells showed similar expression levels and similar GFP fluorescent activity at 37°C. This result substantiates our hypothesis that OmpT protease could be a possible factor responsible for reducing the expression of GFP at 37°C for WT GFP clone in K12 hosts like DH5α, JM109, LE 392 since the levels of GFP expression of SDM II clone in such cells at 37°C is higher than that seen with WT GFP clone at the same temperature.

## Background

The Green Fluorescent Protein (GFP) isolated from coelenterates, such as the Pacific jellyfish, *Aequoria victoria*, has been popularly used as a reporter gene in the determination of gene expression, cell lineage tracer and for protein localization [1,2]. When linked to other proteins as N- and C-terminal fusions, GFP maintains its fluorescent properties in the absence of any substrate or cofactor, hence functions as a convenient fluorescent tag. Several organisms that have been tested successfully for expression of GFP include bacteria, yeast, slime mold, plants, drosophila, zebrafish, and mammalian cells etc [3].

The gene for GFP is coded by 238 amino acids to yield a protein of 27 kDa molecular size. The three amino acids Ser65-Tyr66-Gly67, situated close to the N terminus portion of the GFP molecule, have been documented to function as a fluorophore that is generated by a sequential mechanism in an auto-catalytic process. Gly67 is reported to be essential for the formation of the fluorophore with no substitutions possible [4]. The absorbance/excitation peak of wild type GFP is at 395 nm with a minor peak at 475 nm with extinction coefficients of ~30,000 and 7,000 m^-1 ^cm^-1^. The reaction, which is active in a wide range of pH, is thermosensitive which decreases at temperatures greater than 30°C. However, once expressed, GFP is quite thermostable. It is resistant to denaturation and partial to near total renaturation occurs within minutes following reversal of denaturing conditions by dialysis or neutralization [5].

Random and directed point mutations of gene for GFP have resulted in various changes in its fluorescent behaviour. These include mutation of Tyr66 in the fluorophore to His66 resulting in a shift of the excitation maximum to the UV (383 nm) with emission in the blue at 448 nm [6]. A T66W mutant is blue-shifted albeit to a lesser degree. Mutation of Ser65 to Thr, Ala, Cys or Leu causes a loss of the 395 nm excitation peak with a major increase in blue excitation [7,8]. When combined with Ser65 mutants, mutations at other sites near the fluorophore such as V68L and S72A can further enhance the intensity of green fluorescence produced by excitation at 488 nm. It was shown that the S208L mutation contributes to both a higher intrinsic brightness of GFP and a higher expression level in *E. coli *[9]. Another improvement of GFP expression is by codon optimization [10,11].

Literature reports also suggest chemical agents that lead to loss of GFP fluorescence. GFP readily loses its auto-fluorescence upon exposure to oxyradicals as measured by fluorescence spectroscopy with maximum susceptibility to oxyradical-induced damage at pH 6.5, and least susceptibility at pH 8.5 [12]. Reduction of purified GFP by sodium dithionite results in a rapid loss of fluorescence that slowly recovers in the presence of room air. While insensitive to sulfhydryl reagents such as 2-mercaptoethanol, treatment with the sulfhydryl reagent dithiobisnitrobenzoic acid (DTNB) is known to irreversibly eliminate GFP fluorescence [13].

Truncation of more than seven or nine amino acids from the N and C terminus respectively of GFP leads to total loss of its fluorescence properties [14]. The GFPuv (F99S/M153T/V163A) mutation results in increased fluorescence than wild-type GFP at room temperature. It is proposed that the M153T and V163A mutations in GFPuv may partially account for the increased maturation efficiency in GFPuv since they improve the Arg96-Tyr66 interaction. The same is true for the S147P mutation in S147P-GFP [15].

Due to the higher fluorescent intensity, pGFPuv plasmid carrying a gene coding for GFPuv under lac promoter was chosen for this study. When this plasmid was introduced into competent cells of DH5α and BL21, we noticed variable expression of GFP in both the cell types with relatively higher expression levels in BL21 cells. This result indicated that OmpT protease, which is absent in BL21 cells could be playing a role in inhibiting the proteolysis of GFP resulting in higher GFP fluorescence. Interestingly, Shi and Su [16] have reported that the expression of GFP on the cell surface of *E. coli *is not affected by the OmpT proteases, although the GFP contains two putative OmpT proteolytic sites. In an effort to elevate the GFP expression at 37°C, we decided to mutate the OmpT proteolytic sites of the GFP and were surprised to see a dramatic reduction in the fluorescence when one of the OmpT proteolytic sites close to the fluorophore was mutated whereas the mutation of the second OmpT site caused >250% enhancement in the fluorescent properties of GFP. The results of our experimental findings are discussed in this paper.

## Results

### Construction of pBAD24M-GFPuv (WT), pBAD24M-SDM I and pBAD24M-GFP SDM II

**Figure 1:**
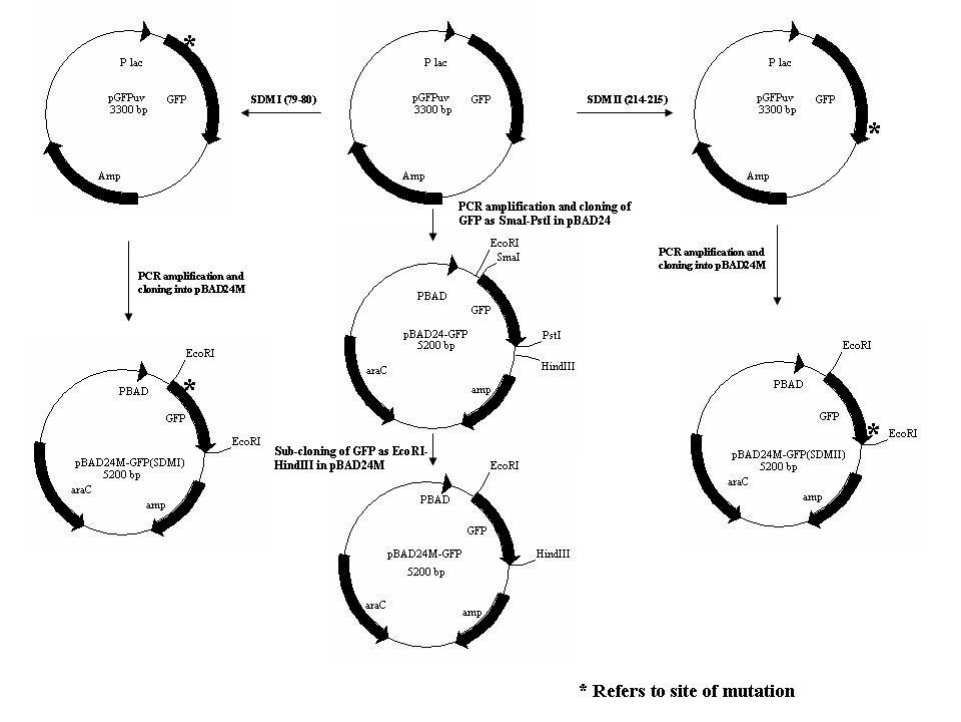
**Schematic representation of construction of pBAD24M-GFP and pBAD24M-GFP SDM I and SDM II mutants**.

Fig. 1 depicts the schematic representation of generation of GFP uv (Wild Type -WT) and various GFP variants under the arabinose promoter. The constructs were confirmed by DNA sequencing to establish the intactness of the gene of GFP along with confirmation of the mutations carried out to generate the OmpT proteolytic site deleted GFP variants.

### GFP fluorescence in *E. coli *K12 and *E. coli *B strains from pBAD24M-GFPuv plasmids at 37°C

**Figure 2:**
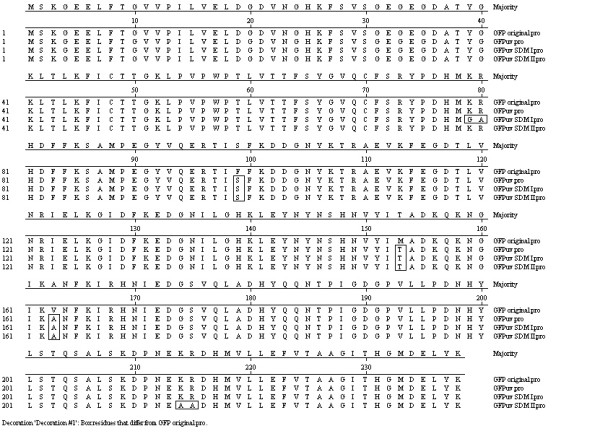
**Amino acid sequences of GFP, GFPuv, mutated GFPuv (K79A/R80A-SDM I) and (K214G/R215A-SDM II)**. The altered sequences are denoted by rectangular boxes.

**Figure 3:**
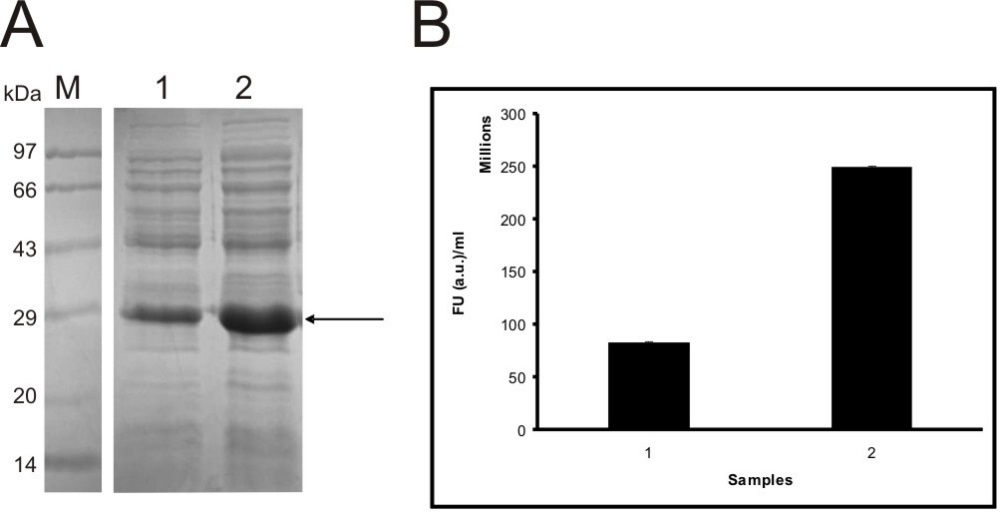
**GFP expression and activity in two different types of *E. coli *hosts**. (A) GFP expression from pBAD24M-GFPuv clone at 37°C. Lane 1: Protein molecular weight marker (14 to 97 kDa); lane 2, GFP expression in DH5α cells; lane 3, GFP expression in BL21 cells. (B) GFP fluorescence units of pBAD24M-GFPuv at 37°C. Sample 1, pBAD24M-GFPuv in DH5α cells; Sample 2, pBAD24M-pGFPuv in BL21 cells.

The alignment of amino acid sequences of the native WT GFP and GFPuv are depicted in Fig. 2. The GFP expressed from a stronger inducible promoter like araBAD showed a high yield of GFP expression in BL21 cells (Fig. 3A, lane 3) as compared to that obtained in DH5α cells (Fig. 3A, lane 2). The fluorescent intensity of GFP also was higher for GFP expressed in BL21 cells (Fig. 3B, sample 2) than in DH5α cells (Fig. 3B, sample 1).

### GFP fluorescence of GFPuv WT and OmpT deleted GFPuv mutants in shake flask studies in *E. coli *DH5α at 30°C and 37°C

**Figure 4:**
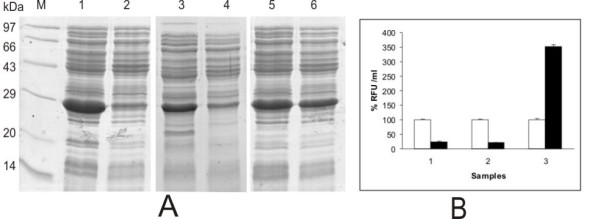
**Effect of induction temperature on GFP expression and fluorescence from various GFP constructs**. (A) SDS-PAGE profile of GFP expressed from pBAD24M-GFP (WT), SDM I and SDM II clones in *E. coli *K12 DH5α cells at 30 and 37°C. Note the enhanced expression of GFP at 30°C for all the clones in comparison to 37°C. Lanes 1, 3, 5 represent GFP expressed at 30°C from WT, SDM I and SDM II clones respectively while lanes 2, 4, 6 represent GFP expressed from WT, SDM I and SDM II at 37°C respectively. (B) Fluorescence activity units of GFP mutants. Sample 1: pBAD24M-GFPuv; Sample 2: SDM I; Sample 3: SDM II. Empty bars represent 30°C induced samples while filled bars represent 37°C induced samples. GFP activity units obtained at 37°C was calculated taking GFP activity units achieved at 30°C as 100%.

The WT GFPuv, SDM I and SDM II GFP mutants showed variable degrees of GFP fluorescence with maximum fluorescence seen with SDM II mutant (Fig. 4B). However, the expression levels of the GFP were more or less similar for all the clones except that the expression levels were higher at 30°C in comparison to 37°C (Fig. 4A).

### GFP fluorescence of GFPuv WT and OmpT deleted GFPuv SDM II in shake flask studies in *E. coli *K12 strains like LE392, JM109 and in *E. coli *BL21 at 30°C and 37°C

**Figure 5:**
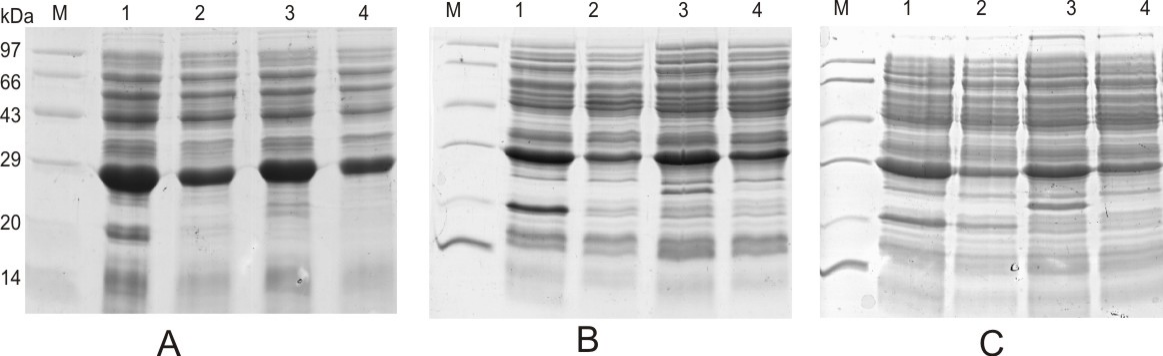
**SDS-PAGE of GFP expression of GFP WT and SDM II in BL21, JM109 and LE392 at 30°C and 37°C**. Panels A, B and C represent GFP expression in BL21, JM109 and LE392 cells respectively. M: Medium molecular weight marker (14-97 kDa); lanes 1 and 2: GFP WT expression at 30°C and 37°C respectively; lanes 3 and 4: SDM II expression at 30°C and 37°C respectively.

**Figure 6:**
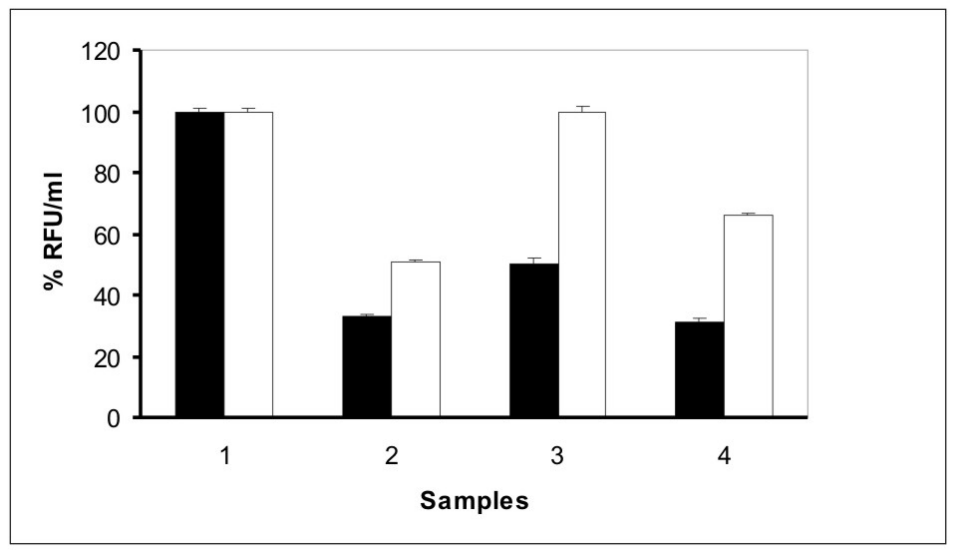
**Fluorescence data of GFP WT and SDM II in BL21, DH5α, JM109 and LE392 at 37°C**. The relative fluorescence values with ± SEM are presented with the fluorescence of GFP obtained in BL21 defined as 100 (arbitrary unit). Sample 1: BL21; sample 2: DH5α; sample 3: JM109; sample 4: LE392. Filled bars represent WT while the empty bars represent SDM II GFP mutant.

The GFP expression was high at 30°C in comparison to 37°C in all the *E. coli *hosts of K12 background while the expression was similar in OmpT deleted host BL21 (Fig. 5) at both the temperatures. The GFP fluorescence measured for all the samples at 37°C showed high activity of the GFP SDM II mutant in comparison to the WT GFP uv (Fig. 6) especially in the OmpT^+ ^hosts.

### Western blot of GFPuv WT and GFP SDM II mutant

**Figure 7:**
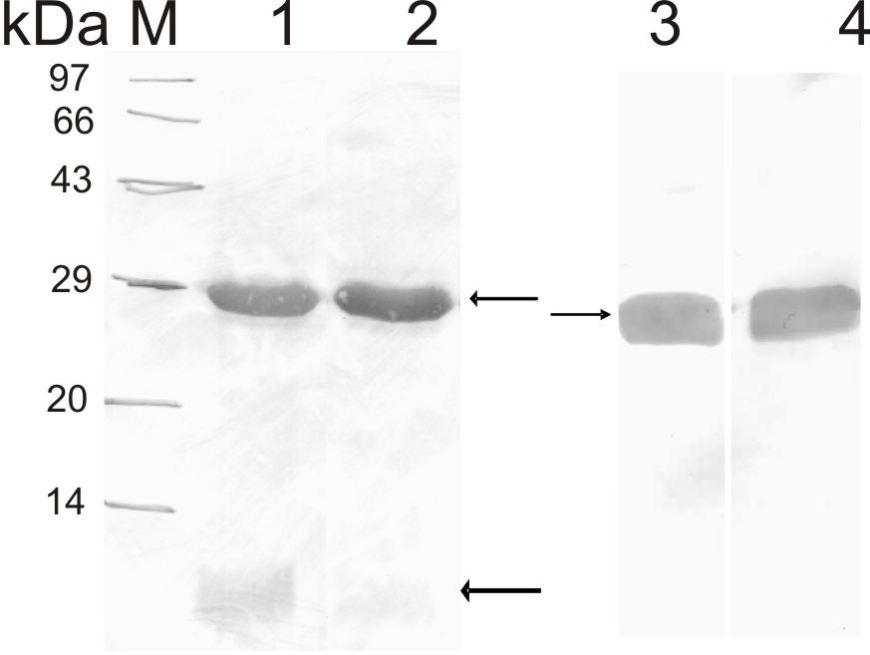
**Western blot of GFP expressed from WT and SDM II clones**. M: Medium molecular weight marker 97-14 kDa); lane 1: Supernatant of WT GFP expressed from JM 109; lane 2: Supernatant of GFP SDM II expressed from JM 109 cells; lanes 3 and 4: WT GFP and SDM II mutant expressed in BL21 cells respectively. The upper arrow shows the intact GFP (29 kDa) while the lower arrow shows the degraded form of GFP (of < 14 kDa) observed only with WT GFP clone and negligible with the GFP SDM II clone.

When the recombinant GFP expressed from the WT and the SDM II mutant in JM 109 cells were blotted with anti-GFP antibody, a prominent degradation of GFP was observed in WT clone while negligible degradation was seen for the GFP SDM II mutant (Fig. 7, lanes 1 and 2). Both these clones did not show any degradation when expressed in an OmpT minus host like BL21 (Fig. 7, lanes 3 and 4).

### Bioreactor studies

**Figure 8:**
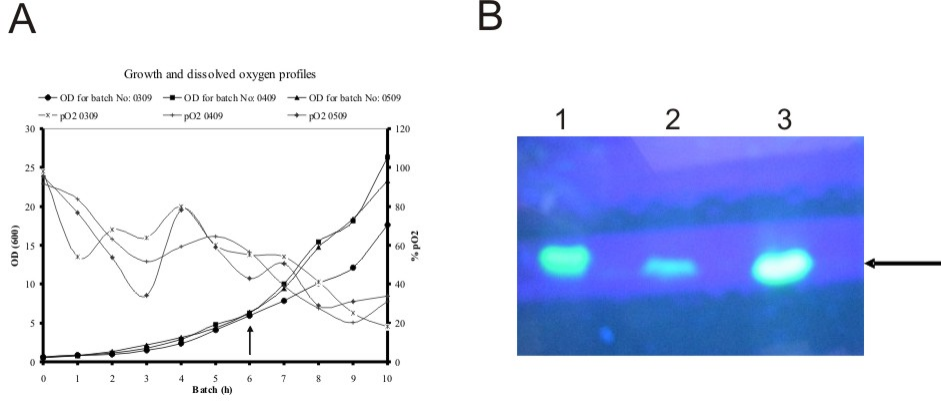
**Fermentation studies of GFP expression**. (A) Growth profiles of pBAD24M-GFP, SDM I and SDM II at 30°C in DH5α cells in a bioreactor. The arrow indicates the point of induction. The growth was monitored at A_600 _nm and DO levels were indicative of effective viable cells. (B) Fluorescence of the GFP samples of harvest samples of pBAD24M-GFPuv (lane 1), SDM I (lane 2) and SDM II (lane 3) under uv light after loading on SDS-PAGE without sample boiling. Arrow indicates the fluorescent protein.

To substantiate the observations of the shake flask experiments, we carried out bioreactor studies of all the three mutants of GFP under similar experimental conditions in *E. coli *K12 host DH5α at 30°C. The growth pattern and the total protein at the time of harvest as depicted in Fig. 8(A) indicated that the amount of GFP expressed in all the three mutants were similar as judged by densitometry scanning (Table 1). It is clear from Fig. 8(B) that the equal protein of the soluble fractions of all the three fermenter runs when analyzed on SDS-PAGE without sample boiling exhibited maximum fluorescence in the SDM II sample indicating the possibility of higher stability of the GFPuv molecule that carried the second OmpT proteolytic site mutation. Interestingly, the specific activity of the GFP expressed from SDM II was nearly four folds higher than the other samples tested (Table 1). It is important to note that SDM I mutation causes nearly 80% loss in specific activity of the GFP fluorescence as seen in Table 1.

**Table 1:** Quantitation of active GFP molecules obtained from different clones of GFPuv from pBAD24M-GFP constructs in DH5α cell free extracts at 30°C.

Clone	Total Protein (mg/ml)^	GFP (mg/ml)*	FU/ml^# ^(× 10^6^)	GFP (mg/ml)^$^	Specific activity (× 10^6^)
**GFPuv (WT)**	9.52	1.02	36.2 ± 4.0	0.39 ± 0.038	3.8
**GFPuv-SDM I**	8.67	1.16	9.3 ± 0.3	0.10 ± 0.002	1.1
**GFPuv-SDM II**	9.92	1.05	139.6 ± 6.0	1.51 ± 0.037	14.1

^Estimation by BCA method

* as judged by densitometry scanning

# The fluorescence units (FU) obtained after an excitation of GFP samples at 488 nm and emission at 507 nm. The values are ± SEM

$ The quantity of recombinant GFP in the sample is determined by comparing its fluorescent reading with that of known GFP standard curve (supplied in the kit). The values are ± SEM

### Comparison of GFP expression from SDM II and native GFPuv in BL21 cells

Both pBAD24M-GFPuv (WT) and pBAD24M-GFPuv (SDM II) plasmids in *E. coli *BL21 cells showed similar expression levels and also similar GFP fluorescent activity at 37°C (3185710 FU/ml and 3485710 FU/ml respectively). This result is encouraging and proves our hypothesis that OmpT is the possible responsible factor for reducing the expression of GFP at 37°C for WT GFP in K12 hosts since the levels of GFP expression of SDM II clone in DH5α cells at 37°C is much higher than that seen with WT GFP clone at the same temperature.

### Effect of inclusion of OmpT inhibitor like zinc on GFP fluorescence in crude cell lysates of *E. coli *k12 expressing WT GFP

**Figure 9:**
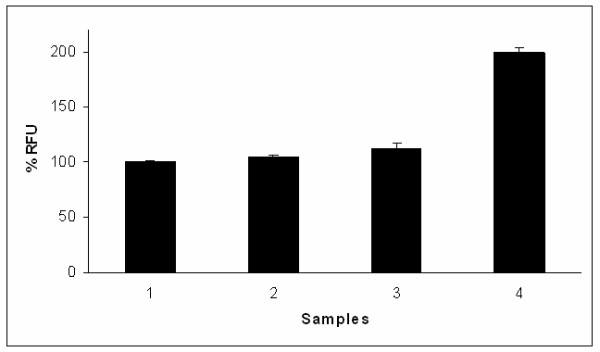
**Effect of OmpT inhibitor like zinc on GFP fluorescence activity**. The GFP fluorescence observed with the WT GFP clone was taken as 100% and served as a control for this assay. The GFP fluorescence activity was found to be greater in samples with zinc in the reaction assay. Note that 3 mM zinc dramatically reduced the extent of damage to GFP by showing highest GFP fluorescence activity.

The GFP expressed in DH5α cells (OmpT^+ ^host) when incubated with varying concentrations of zinc showed more GFP fluorescence activity units than the control without zinc. The data represented in Fig. 9 clearly demonstrates that presence of OmpT in the crude cell lysates of K12 host plays a role in reducing GFP fluorescence since the well known OmpT inhibitor like zinc improved GFP fluorescence activity units from the same lysate.

## Discussion

When we investigated the expression of GFP as a model foreign protein in two commonly employed bacterial hosts for expression of heterologous proteins namely BL21 and DH5α, we noticed two interesting observations. Firstly, the GFP was more fluorescent at 30°C over 37°C and secondly, the GFP expression was more predominant in BL21 cells than DH5α. To confirm our observations, we tested GFP expression in another OmpT^+ ^host, namely LE392 and found that the GFP fluorescence was reduced to a large extent when the GFP induction was carried out at 37°C in comparison to 30°C. Although Seo et al., [17] suggest that increased stress levels in cloning strains like JM109, HB101 result in low expression of GFP in comparison to lesser stress level *E. coli *host BL21 that is lon^- ^and OmpT^- ^[18], we interpreted this result from another angle. A close look into the phenotype of these two hosts indicates the presence of a OmpT protease gene in DH5α cells that is deleted in BL21. To address the possibility of low expression of GFP in *E. coli *K12 hosts due to degradation of the GFP by the host OmpT protease, we undertook mutation of the putative OmpT proteolytic sites of the GFPuv and studied its effect on GFP expression and fluorescence.

Proteases play an important regulatory role in the degradation of abnormal proteins and in the response to stressful conditions in *Escherichia coli *[19]. Goldberg et al. [20] estimated that 5 to 12 percent of the total cellular protein is degraded per hour in non-growing cells. Interestingly, induction of a recombinant protein elicits a stress response which includes increased protease activities [21]. Some of these proteases were found to have both *in vivo *and *in vitro *activity towards misfolded or unfolded polypeptides, which can arise when overproduced in *E. coli *[22]. As a result, the induction and over expression of a recombinant protein can lead directly to increased proteolysis.

OmpT protease degrades recombinant proteins *in vivo *[23] and retains significant activity under extremely denaturing conditions [24] and heat shock [25] is a surface membrane serine protease of the omptin family of gram-negative bacteria [26] shown to cleave several antimicrobial peptides, activate human plasminogen, and degrade some recombinant heterologous proteins [27,28]. As a result, OmpT gene with respect to GFP expression was studied in detail in this article.

GFP is reported to have two putative OmpT proteolytic sites at K79R80 and K214R215. When we mutated the first OmpT site to K79G and R80A respectively (SDM I), we noticed loss of nearly 70% GFP fluorescence. Our result correlated with the observations of Li et al. [3] who have shown that substitution of amino acids between the regions 76 to 81 of gene for GFP causes complete loss of fluorescence since the helix formed in this region is required for fluorescence. Our present observations of nearly 30% residual fluorescent activity after the modification of 79 and 80 amino acids actually point out for the first time that K79 and R80 in this helix are crucial for the fluorescent behaviour of GFP.

OmpT expression has been reported to be minimal at 30°C as suggested by Stathopoulos et al. [29] and hence our findings of high amounts of GFP expression at 30°C over 37°C in DH5α, JM109 and in LE392 cells could be attributed to the OmpT activity in both the K12 hosts. Moreover, since a recent report of Choy et al. [30] suggests that Lon protease is inactive on purified GFP, the contribution of lon protease for reduced GFP fluorescence in these cell lines appear remote.

The observations of nearly 250% increased expression of the SDM II GFP mutants possibly indicate the enhanced stability of the GFP achieved after deletion of the OmpT site thereby reducing its degradation. It is important to emphasize at this stage that the SDM II GFP mutant described in this report, has an intact first OmpT proteolytic site and this could be responsible for the observed minor degradation of GFP in the SDM II mutant in the western blot.

The enhanced fluorescence of SDM II GFPuv mutant actually indicates a highly stable configuration of the GFP molecule. It is necessary to emphasize that this region falls in the 11^th ^beta sheet of the GFP molecule and this is the first report of enhanced GFP fluorescence with respect to the mutation of amino acids at 214 and 215 positions. Mutation of S208F and T217A has been tested and shown to result in yellow and red fluorescence [31] and hence the mutations of K214A and R215A described in this work now opens up new exploring possibilities of using this mutant GFP for further studies.

BL21 was developed as a production *E*. *coli *strain through removal of proteases OmpT [18]. Seo et al. [17] show that BL21 exhibits lowest cellular stress levels at 30°C and hence expression of foreign proteins is highest in these cells as compared with other *E*. *coli *strains like JM109, HB101 etc. Our data on enhanced levels of GFP fluorescence of SDM II mutant over native GFPuv clone in three OmpT^+ ^hosts namely DH5α, JM109 and LE392 at 37°C reiterates the role played by host OmpT in determining differences in expression of the proteins in addition to the stress related phenomenon.

The excitation and emission spectra for the SDM II GFPuv protein is nearly identical to wild-type GFPuv (data not shown). When introduced into *E. coli *K12 cells, greater fluorescence was observed for the GFP compared to the native GFPuv, implying that GFP is 'brighter' because more of it is present in a stable, better refolded and functional form. Such highly fluorescent mutants of GFP are well known [32].

Literature reports on susceptibility of GFP towards proteases are varied and not very conclusive. GFP is reported to be highly resistant to proteolysis and is reported to be uncleaved even after prolonged incubation with trypsin or pronase despite having several putative tryptic and chymotryptic sites in its exposed loops [33]. Such a GFP molecule has been artificially rendered sensitive to proteolysis by inserting five amino acids, IEGRS, in loops at position 157, 172, or 189 where Chiang et al. [33] suggest that while trypsin cleaved the native (folded) form of each mutant at a unique site defined by the insert, pronase also yielded similar digestion patterns in these variants. However, further proteolysis was also observed, suggesting that the primary cleavage relaxes GFP structure and reveals previously inaccessible sites. It is tempting to speculate that these inaccessible sites could be even the OmpT proteolytic sites that are present in the coding gene for GFP.

Although the GFP described in this study is not exported into the surface of the cell, due to lack of any signal sequence in the GFP constructs made, one might wonder as to how the OmpT residing on the outer surface of the bacterial cell, would be accessible for degradation of GFP that is localized in the cytoplasm of the cell. The GFP degradation in the present study was observed only with the *E. coli *induced cell lysates (containing free LPS and OmpT) and not with the whole bacterial cells. The fact that OmpT is known to exert its proteolytic activity only in the presence of lipopolysaccharides [34] in both the extracellular and periplasmic space [35], of *E. coli *prompts us to suggest that OmpT might be the contributory factor for the observed GFP degradation in the WT clone at 37°C. Interestingly enough, OmpT has been found to be present even in plasmid DNA preparations prepared from DH5α cell lysates that is capable of cleaving proteins like Cyclin A as suggested by Yam et al. [36]. This report also emphasizes the robust nature of OmpT that is resistant to heat, boiling, alkali treatment, ethanol treatment, DNase treatment. The method of preparation of the cell lysate described by Yam et al. [36] is similar to that we have followed and hence, the possibility of having OmpT in the soluble fraction of cell (comprising of both periplasmic and cytoplasmic fractions) is the only reason that we could attribute to the GFP degradation observed in our present study. Our data on greater GFP fluorescence activity in crude cell lysates of K12 hosts expressing WT GFP in presence of a known OmpT inhibitor like zinc [24] reiterates the role played by OmpT in the context of GFP fluorescence.

Hence the possible effect of the OmpT in the preparation of new GFP constructs described here could prove useful for identifying germ layer cells (endodermal, ectodermal, and mesodermal), as well as neuronal, haematopoietic, endothelial, and cartilage cells, and also provided a useful battery of tissue/receptor-specific screening assays for new chemical entities. Since the emitted fluorescence is non-toxic and shows enhanced fluorescence at 37°C, this construct would prove beneficial for using fluorescence-activated cell sorting (FACS), thus avoiding any staining procedure, expensive mRNA analysis or hazardous radiolabeled binding assays.

## Materials and methods

### Strains, plasmids and culture conditions

pBAD24 was a gift from M. Yasuda, Institute of Genetics, Japan. pGFPuv plasmid was purchased from Stratagene, USA. *Escherichia coli *K12 DH5α, LE392, JM109 were purchased from Bangalore Genei Pvt Ltd, Bangalore, India while BL21 was from Stratagene, USA respectively. All chemicals and oligos were from Sigma, USA. While the restriction enzymes were from Bangalore Genei Pvt. Ltd, Bangalore, India, the GFP quantitation kit was from Cell Biolabs, USA. Micro BCA kit for protein estimations was procured from Pierce, USA. The polyclonal rabbit anti-GFP antibody was procured from Calbiochem, USA.

### GFPuv fluorescence from pGFPuv plasmid in *E. coli *K12 and *E. coli *B strains

pGFPuv plasmid carries the gene coding for GFP under lac promoter [37]. This plasmid was introduced into two types of *E. coli *cells namely *E. coli *K12 DH5α and *E. coli *BL21 and GFP fluorescence was examined with arabinose as an inducer at two different temperatures viz 30 and 37°C.

### Cloning of GFPuv in pBAD24M vector and expression studies

Since pBAD24M vector could be easily used for hyper expression of heterologous proteins in *E. coli *K12 host due to the presence of enhancer elements as described before [38], we decided to clone the gene coding for GFPuv into this vector and examine if the difference in the GFP fluorescence was due to difference in the amount of GFP expression per cell rather than the stability of the protein. The GFPuv was PCR amplified from pGFPuv vector using GFP specific primers and cloned into EcoR1/HindIII sites as described earlier by Banerjee et al. [38]. This plasmid was introduced into competent cells of *E. coli *K12 and *E. coli *B strains and GFP expressions were carried out in plain Luria Bertani broth (LB). The cells were grown till it reached OD_600 _of 0.5-0.6 and then induced with L+ arabinose at 13 mM concentration. The induction was carried out for 4 h at 30 and 37°C. The samples were later analyzed on SDS-PAGE.

### Comparison of GFP expression and fluorescence of WT and SDM II in *E. coli *OmpT^+ ^and OmpT^- ^hosts

The WT GFP and SDM II GFP mutant were introduced into competent cells of various *E. coli *K12 hosts like JM109, DH5α and LE392 and *E. coli *B host like BL21 and inductions were carried out at 30 and 37°C in two separate flasks with 13 mM arabinose for 4 h as described earlier. The induced cells were subjected to lysis using a homogenizer and the soluble fraction of the cell was taken for SDS-PAGE and fluorescence measurement.

### Site directed mutagenesis (SDM) of K79G and R80A (SDM I)

The forward and reverse primers used for SDM of K79G/R80A were 5'-TAT CCG GAT CAT ATG **GGA GCT C**AT GAC TTT TTC AAG -3' and 5'-CTT GAA AAA GTC AT**G AGC TC**C CAT ATG ATC CGG ATA-3' respectively. SDM was performed following manufacturer's protocol of site directed mutagenesis kit (Stratagene, USA) using pGFPuv plasmid as the template. SDM I was confirmed with incorporation of SacI site for identification of mutated plasmid.

### SDM of K214A and R215A (SDM II)

The forward and reverse primers used for SDM of K214A/R215A were 5'-AAA GAT CCC AAC GAA G**CA GCT G**AC CAC ATG GTC CTT -3' and 5'-AAG GAC CAT GTG GT**C AGC TG**C TTC GTT GGG ATC TTT-3' respectively, following manufacturer's protocol of site directed mutagenesis kit (Stratagene, USA) using pGFPuv plasmid DNA as the template. SDM II was confirmed with incorporation of Pvu II site for identification of the mutated plasmid.

### Subcloning of GFP (SDM I) and GFP (SDM II) into pBAD24M vector

GFPuv (SDM I) was PCR amplified using pGFPuv SDM I plasmid as template. The primers used were forward primer as 5'-CCG CCG GAA TTC GAT ATC ATG AGT AAA GGA GAA GAA CTT TTC -3' and reverse primer as 5'-CCG CCG GAA TTC TTA TTT GTA GAG CTC ATC CAT GCC -3'. The PCR product was cloned into pBAD24M vector [38] at the EcoRI site. The correct orientation of clones was confirmed by EcoRV digestion and tested for expression in suitable *E. coli *host. Similarly GFP SDM II was subcloned into pBAD24M vector.

### Comparison of GFP expression from SDM II and native GFPuv in BL21 cells

Both pBAD24M-GFPuv and pBAD24M-GFPuv (SDM II) plasmids were introduced into *E. coli *BL21 competent cells and the cells were induced with 13 mM arabinose at 37°C for 4 h and after lysing the induced cell pellets, the supernatants were checked for GFP expression using the fluorescence assay kit.

### Western blot

Protein samples were separated on 15% SDS-PAGE gels and electroblotted onto nitro cellulose membrane. The membrane was blocked with 3% BSA in TBST (10 mM Tris.Cl with 150 mM NaCl and 0.1% Tween 20) for 2 h at room temperature. The membrane was then incubated for 2 h at room temperature with rabbit anti-GFP antibody (diluted in TBST with 0.3% BSA). After three washes with TBST buffer, the membrane was incubated for 1 h with alkaline phosphatase conjugated anti rabbit IgG (diluted in TBST with 0.3% BSA). The membrane was washed three times and specific protein was visualized by adding BCIP/NBT solution (Bangalore Genei, India).

### Bioreactor studies, cell fractionation and SDS-PAGE analysis

#### Fermentation parameters

Modified LB of the following composition (24 g/L yeast extract, 12 g/L tryptone and 10 g/L sodium chloride) was used as the fermentation medium. The feed medium comprised of 240 g/L yeast extract, 120 g/L tryptone and 10 g/L sodium chloride with 100 μg/ml ampicillin. Fermentation process was carried out in a fed batch mode for 10 h at 30°C where the process was initiated by adding 100 ml of overnight culture (in LB) to 900 ml of the fermentation medium. The pH was maintained at 7.0 by automatic addition of either 12% ammonia solution or 30% O-phosphoric acid. The aeration was controlled at 1 vvm and the agitation was gradually increased from 300 to 800 rpm to maintain the dissolved oxygen level above 30%. The culture was induced with 3 mM L (+) arabinose at an OD of 6.0 and the feed was added at the rate of 30 ml/h after 30 minutes of induction.

#### Culture harvest and cell disruption

The fermentation broth was centrifuged at 12,500 g for 10 minutes at 4°C and the induced cell pellet was re suspended in 10 mM Tris-HCl, pH 8.0 at an OD of 50. This suspension was subjected to cell disruption using a high pressure homogenizer (M/s Niro Soavi, Italy). Cell disruption was carried out at 800 to 900 bars for two passages. The homogenized cell lysate was centrifuged at 12,500 g for 15 minutes at 4°C to separate the soluble and the insoluble fraction. The presence of expressed GFP protein (~27 kDa molecular size) was analyzed on 15% SDS-PAGE.

Cell growth was measured by monitoring attenuance (*D*) at 600 _nm _on a UV/visible spectrophotometer and total protein was estimated using the micro BCA kit.

#### Measurement of GFP fluorescence

GFP fluorescence for the fermentation samples was done as per the protocol described by Baird et al. [39]. GFP quantitation fluorometric kit that measures GFP fluorescence in a fluorometer was also used for exact quantitation of GFP produced. The quantity of GFP in sample was determined by comparing its fluorescence reading with that of known recombinant GFP standard curve. The kit has a detection sensitivity limit of 100 ng of GFP/ml. A proprietary GFP quench solution was also included for determining autofluoresence of cell sample.

For most of the experiments, unless mentioned otherwise, the fluorescence was measured in triplicate from cultures induced at 30°C and 37°C after cell density normalization. The supernatants obtained after lysing the induced cells of the same optical density using a homogeniser served as the source for GFP fluorescence assay. The relative fluorescence values are presented with the fluorescence of GFP obtained in BL21 defined as 100 (arbitrary unit).

The WT GFP was expressed in DH5α cells following the protocol described in the manuscript. The GFP expressing cells were lysed by sonication and the supernatant was used as GFP source for the inhibitor experiment.

#### Effect of inclusion of OmpT inhibitor like zinc on GFP fluorescence in crude cell lysates of *E. coli *K12 expressing WT GFP

200 μl of the crude DH5α lysate (OmpT^+ ^host) carrying GFP protein (total protein of 4 mg/ml) was incubated with varying concentrations of zinc acetate (0.5, 1.0 and 3 mM) and the contents were incubated at 37°C in a water bath for 16 h. A control tube without zinc was also kept under similar experimental conditions. All the samples were pelleted to remove any precipitated material and the supernatants were analyzed for protein estimation by BCA method. Equal amounts of protein were taken for the GFP fluorescence assay to examine the amount of GFP fluorescence in all the samples.

## Competing interests

The authors declare that they have no competing interests.

## Authors' contributions

SSS carried out all the cloning experiments described in this paper along with preliminary drafting of the manuscript. The bioreactor studies along with SDS-PAGE analysis and protein estimations were carried out by the upstream process development team comprising SR, PK and AK. The GFP assay of all the samples and analysis of all the results was done by VAR. The concept of role of possible OmpT cleavage sites on GFP fluorescence was conceived by SP. The drafting of the manuscript along with supervision of all the experiments was done by SP. All the authors have read and approved the final version of the manuscript.
